# Effect of EGFR-TKIs combined with craniocerebral radiotherapy on the prognosis of *EGFR*-mutant lung adenocarcinoma patients with brain metastasis: A propensity-score matched analysis

**DOI:** 10.3389/fonc.2023.1049855

**Published:** 2023-02-09

**Authors:** Guangchuan Deng, Xiaojing Tan, Yankang Li, Yingyun Zhang, Qi Wang, Jianbin Li, Zhenxiang Li

**Affiliations:** ^1^ School of Graduate Studies, Shandong First Medical University and Shandong Academy of Medical Sciences, Jinan, China; ^2^ Department of Radiation Oncology, Shandong Cancer Hospital and Institute, Shandong First Medical University and Shandong Academy of Medical Sciences, Jinan, China; ^3^ Department of Oncology, Dongying People’s Hospital, Dongying, China

**Keywords:** Adenocarcinoma of lung, brain neoplasms, carcinoma, non-small-cell lung, progression-free survival (PFS)

## Abstract

**Background and Purpose:**

Epidermal growth factor receptor (EGFR)-mutant lung cancers are associated with a high risk of developing brain metastases (BM). Craniocerebral radiotherapy is a cornerstone for the treatment of BM, and EGFR-TKIs act on craniocerebral metastases”. However, whether EGFR-TKIs combined with craniocerebral radiotherapy can further increase the efficacy and improve the prognosis of patients is unclear. This study aimed to evaluate the difference in efficacy between targeted-therapy alone and targeted-therapy combined with radiotherapy in EGFR-mutant lung adenocarcinoma patients with BM.

**Materials and Methods:**

A total of 291 patients with advanced non-small cell lung cancer (NSCLC) and *EGFR* mutations were enrolled in this retrospective cohort study. Propensity score matching (PSM) was conducted using a nearest-neighbor algorithm (1:1) to adjust for demographic and clinical covariates. Patients were divided into two groups: EGFR-TKIs alone and EGFR-TKIs combined with craniocerebral radiotherapy. Intracranial progression-free survival (iPFS) and overall survival (OS) were calculated. Kaplan–Meier analysis was used to compare iPFS and OS between the two groups. Brain radiotherapy included WBRT, local radiotherapy, and WBRT+Boost.

**Results:**

The median age at diagnosis was 54 years (range: 28–81 years). Most patients were female (55.9%) and non-smokers (75.5%). Fifty-one pairs of patients were matched using PSM. The median iPFS for EGFR-TKIs alone (n=37) and EGFR-TKIs+craniocerebral radiotherapy (n=24) was 8.9 and 14.7 months, respectively. The median OS for EGFR-TKIs alone (n=52) and EGFR-TKIs+craniocerebral radiotherapy (n=52) was 32.1 and 45.3 months, respectively.

**Conclusion:**

In *EGFR*-mutant lung adenocarcinoma patients with BM, targeted therapy combined with craniocerebral radiotherapy is an optimal treatment.

## Introduction

Lung cancer is the leading cause of morbidity and mortality around the globe (1, 2). According to the International Agency for Research on Cancer, 2.2 million new cases of lung cancer were reported worldwide in 2020, accounting for 11.4% of newly reported cancer cases (IARC Biennial Report 2020-2021, World Cancer Report 2020). Non-small-cell lung cancer (NSCLC) accounts for approximately 85% of all lung cancers and most patients are diagnosed at an advanced stage (3). Patients with advanced NSCLC have been treated with individualized molecular-targeted therapy based on the profiles of driver gene alteration, among which mutant epidermal growth factor receptor (*EGFR*) is an important therapeutic target (4). Brain metastases, the most common site of distant metastasis in NSCLC, is the main cause of poor prognosis (5, 6). Some studies have shown that the incidence of brain metastases in lung adenocarcinoma patients with EGFR mutations is slightly higher than that in patients with wild-type EGFR, with an approximate incidence rate of 60% (7–9), indicating that lung adenocarcinoma patients with EGFR mutations may be more prone to developing brain metastases. With advancements in systemic treatment and radiotherapy techniques, the overall survival (OS) of patients has been further prolonged from 3–6 months to 19–58 months, increasing the risk of developing brain metastases during the entire disease course (10–13). The two aforementioned reasons cause a high incidence of brain metastases in *EGFR*-mutant patients with lung adenocarcinoma.

In clinical practice, targeted-therapy and radiotherapy are the main treatment options for *EGFR*-mutant lung adenocarcinoma patients with brain metastases (9, 14). Such a combination has proven to be effective and safe for brain metastases also from other cancers (15).The BRAIN clinical trial showed EGFR-tyrosine kinase inhibitor (TKI) alone was associated with a significantly longer intracranial progression-free survival (iPFS) in patients with craniocerebral metastasis. This clinical trial showed that a median iPFS of 10.0 months (95% confidence interval [CI], 5.6–14.4) in the icotinib group and 4.8 months (95% CI, 2.4–7.2) in the craniocerebral radiotherapy group (14). The results showed that although EGFR-TKIs alone were effective, their therapeutic effect was limited. Craniocerebral radiotherapy is considered a cornerstone of the treatment of brain metastases (16–18). Craniocerebral radiotherapy can destroy the blood–brain barrier, increasing the absorption of chemotherapy or targeted drugs to a great extent, which provides a theoretical basis for the combination of EGFR-TKIs and radiotherapy (19–21). Moreover, some retrospective studies have shown that craniocerebral radiotherapy combined with EGFR-TKIs is more effective than EGFR-TKIs alone (22, 23). However, certain factors that affect the prognosis of patients, such as age, Karnofsky performance status, extracranial metastasis, and number of brain metastases, were not considered in those studies. Studies have shown that these factors are closely related to the prognosis of patients, which may adversely affect the results and conclusions; therefore these studies cannot be used to judge whether EGFR-TKIs combined with craniocerebral radiotherapy indeed increase the efficacy and improve the prognosis of patients (12, 24). The above factors that affect the prognosis of patients with brain metastases should be considered when formulating a treatment plan, especially when radiotherapy is considered.

We performed this retrospective study to eliminate the impact of potential confounding factors on the results to explore the necessity of craniocerebral radiotherapy for *EGFR*-mutant lung adenocarcinoma patients with brain metastases used propensity score matching (PSM).

## Methods and materials

### Patient cohort

We screened lung cancer patients diagnosed with *EGFR*-mutant lung adenocarcinoma and brain metastases at our hospital between September 2008 and September 2020. The inclusion criteria were as follows: pathologically diagnosed with primary lung adenocarcinoma; mutations in *EGFR* exons 18, 19, or 21; diagnosis of brain metastases using enhanced computed tomography (CT) or magnetic resonance imaging (MRI); detailed clinical information, including treatment options and clinicopathological features; EGFR-TKIs administered (e.g., gefitinib, erlotinib, or icotinib); and no other primary malignancies. Patients with incomplete medical records or those who failed to meet the aforementioned criteria were excluded from the study. The study was approved by the Ethics Committee of Shandong Cancer Hospital and Institute and was conducted in accordance with the Declaration of Helsinki. Patient records were anonymized and identified before data analysis.

The following characteristics were included in the analysis: age, sex, smoking history, *EGFR* mutation status, tumor-node-metastasis (TNM) stage, treatment scheme that included chemotherapy regimens or EGFR-TKI regimens, and information on craniocerebral radiotherapy. Information on the start date of EGFR-TKIs administration, intracranial progression, most recent follow-up, and death were also recorded. Intracranial progression was defined as radiographic progression of pre-existing brain metastases, development of new brain metastases, or both. iPFS was measured and reviewed at the last follow-up visit. The time to intracranial progression was calculated from the start of EGFR-TKIs or craniocerebral radiotherapy to the date of intracranial progression or death. PFS was defined from the date of the start of EGFR-TKIs or craniocerebral radiotherapy to the date of progression or death. OS was calculated from the date of the pathological diagnosis of lung adenocarcinoma to the date of death or re-examination at the last follow-up.

### *EGFR* genotyping

Genomic DNA was extracted from tumor tissues obtained by fiberoptic bronchoscopy or puncture biopsy. Circulating tumor DNA was isolated from the blood and was purified. *EGFR* mutations were detected using next-generation sequencing, droplet digital polymerase chain reaction, or amplification refractory mutation system-PCR. However, owing to the lack of clinical data of some patients, the specific mutational site of EGFR could not be identified.

### Radiotherapy

Brain radiotherapy included whole brain radiotherapy (WBRT), local radiotherapy, and WBRT+Boost. The median prescribed dose was 40 Gy (range, total dose: 30–50 Gy, 2-3Gy/per day, 10-25 fractions) and 50 Gy (range, total dose:30–60 Gy, 2-5.5Gy/per day, 10-30 fractions) in patients treated with WBRT and local radiotherapy, respectively. Additionally, in the WBRT+Boost group, the median prescription dose of the whole brain was 37.5 Gy (range, 30–40 Gy, 2-3Gy/per day, 10-20 fractions). Lastly, the median dose of additional radiation boost for local metastases was 12 Gy (range, 7.2–20 Gy, 3 to 10 fractions). The radiation dose was 30 to 60 Gy in 15 to 30 fractions, 2 to 3 Gy per-fraction. In our study, the number of sequential to the principal WBRT course is 46 (52.9%), and the concomitant (integrated) is 41 (47.1%).

### Statistical analysis

We used the 1:1 PSM method in SPSS Version26.0 to decrease the effect of potential confounding factors between the two groups to eliminate the influence of these factors on the results. The characteristics of the patients before and after pairing were compared using the Chi-squared test. The Kaplan-Meier method was used for survival analysis, and the log-rank test was used to test the influence of individual variables on survival. Statistical significance was set at *P*-values<0.05 (two-sided). Statistical analyses were performed using GraphPad Prism version 8.0.1.

## Results

### Patient characteristics

A total of 291 *EGFR*-mutant lung adenocarcinoma patients with brain metastases were enrolled in the study, with 61 patients in the EGFR-TKI alone group and 230 in the EGFR-TKI+craniocerebral radiotherapy group. The median age of the patients at diagnosis was 55 years (range, 23–81 years). Most patients were female (62.9%) and non-smokers (77.3%). Moreover, mutations in *EGFR* exons 19 and 21 were detected in 41.9% (122/291) and 49.1% (143/291) of patients, respectively. Most of the Lung-molGPA scores of the patients were in the 2.5–4 group (167,57.4%). **Table 1** shows the clinical features of the two groups.

**Table 1:** Clinical features of patients in the EGFR-TKI alone group and EGFR-TKIs combined with cerebral radiotherapy group with chi-square test for Categorical Variables.

Characteristics	Before propensity score matching			After propensity score matching		
	EGFR-TKIs combined with cerebral radiotherapy group (n=230)	EGFR-TKI alone group (n=61)	P value	EGFR-TKIs combined with cerebral radiotherapy group (n=51)	EGFR-TKI alone group (n=51)	P value
Age, years	54 (32-81)	57 (23-81)		57 (37-81)	56 (23-77)	
>60	66 (28.7)	28 (45.9)	0.0106	17 (33.3)	22 (43.1)	0.3083
≤60	164 (71.3)	33 (54.1)		34 (66.7)	29 (56.9)	
Sex						
Female	148 (64.3)	35 (57.4)	0.3164	27 (52.9)	30 (58.8)	0.5497
male	82 (35.7)	26 (42.6)		24 (47.1)	21 (41.2)	
Smoking Status						
Never	179 (77.8)	46 (75.4)	0.6887	38 (74.5)	39 (76.5)	0.8179
Former/current	51 (22.2)	15 (24.6)		13 (25.5)	12 (23.5)	
EGFR Mutation						
Exon 18	5 (2.2)	1 (1.6)	0.6539	0 (0)	0	1
Exon 19	94 (40.9)	28 (45.9)		23 (45.1)	23 (45.1)	
Exon 21	113 (49.1)	30 (49.2)		24 (47.1)	25 (49.0)	
Unclear	18 (7.81)	2 (3.3)		4 (7.8)	3 (5.9)	
Systemic Therapy						
First-line EGFR TKIs	114 (49.6)	44 (72.1)	0.0017	29 (56.9)	36 (70.6)	0.1494
Second-line EGFR TKIs	116 (50.4)	17 (27.9)		22 (43.1)	15 (29.4)	
Lung-molGPA						
1-2	106 (46.1)	18 (29.5)	0.0262	12 (23.5)	13 (25.5)	0. 9432
2.5-3	92 (40.0)	36 (59.0)		32 (62.8)	32 (62.7)	
3.5-4	32 (13.9))	7 (11.5)		7 (13.7)	6 (11.8)	

**Figure 1:**
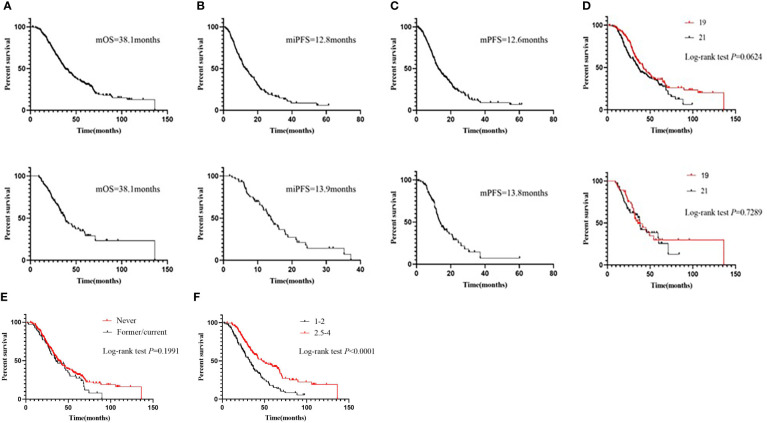
Before PSM: **(A)** Overall survival (OS) of the entire cohort. **(B)** Intracranial progression-free survival (iPFS) and **(C)** progression-free survival (PFS) of the entire cohort. OS of patients stratified according to **(D)** *EGFR* mutation status.After PSM: **(A)** Overall survival (OS) of the entire cohort. **(B)** Intracranial progression-free survival (iPFS) and **(C)** progression-free survival (PFS) of the entire cohort. OS of patients stratified according to **(D)** *EGFR* mutation status. OS of patients stratified according to **(E)** Smoking status; OS of patients stratified according to **(F)** Lung-molGPA.

As of March 2022, 195 deaths (67.0%) were recorded. The median follow-up duration was 63.8 months (interquartile range, 40.3–87.5). The median OS was 38.1 months, the median iPFS was 12.8 months, while the median PFS was 12.6 months (**Figures 1A–C**). There was no difference in OS between the groups with mutations in exons 19 and 21 (41.7 months vs. 36.0 months; log-rank *P*=0.0624; hazard ratio [HR], 0.7510; 95% CI, 0.5557–1.015; **Figure 1D**). There was also no difference in OS between the smoker and non-smoker groups (35.8 months vs. 38.3 months; log-rank *P*=0.1991; HR, 0.7952; 95% CI, 0.5604–1.128; **Figure 1E**). However, the median OS time of the groups with Lung-molGPA score 1–2 and Lung-molGPA score 2.5–4 was 32.3 months and 48 months, respectively (log-rank *P*<0.0001; HR, 2.024; 95% CI, 1.502–2.727; **Figure 1F**).

### Survival analysis before PSM

**Figure 2:**
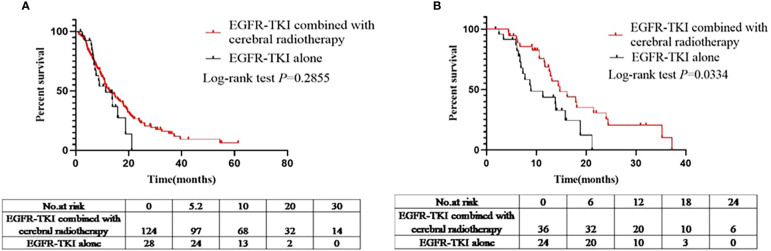
**(A)** Comparison of iPFS between the EGFR-TKIs combined with cerebral radiotherapy group and EGFR-TKI alone group before PSM. **(B)** Comparison of iPFS between the EGFR-TKIs combined with cerebral radiotherapy group and EGFR-TKI alone group after PSM.

A total of 152 patients had intracranial progression after being administered EGFR-TKIs (EGFR-TKIs+craniocerebral radiotherapy group, 124; EGFR-TKI alone group, 28). The iPFS analysis was compared between the EGFR-TKI+craniocerebral radiotherapy and EGFR-TKI alone groups. The median iPFS in the EGFR-TKIs+craniocerebral radiotherapy group and EGFR-TKI alone group was 12.8 and 11.3 months, respectively (**Figure 2A**); there were no significant differences in the median iPFS between the two groups (log-rank *P*=0.2855; HR, 1.376; 95% CI, 0.7661–2.472; **Figure 2A**).

**Figure 3:**
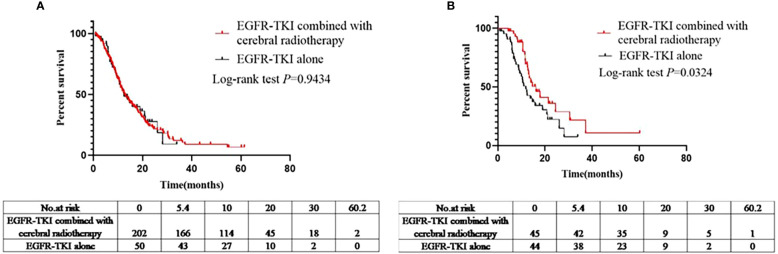
**(A)** Comparison of PFS between the EGFR-TKIs combined with cerebral radiotherapy group and EGFR-TKI alone group before PSM. **(B)** Comparison of PFS between the EGFR-TKIs combined with cerebral radiotherapy group and EGFR-TKI alone group after PSM.

A total of 252 patients showed disease progression after being administered EGFR-TKIs (EGFR-TKIs+craniocerebral radiotherapy group, 202; and EGFR-TKI alone group, 50). Similarly, PFS analysis was performed in the EGFR-TKI+craniocerebral radiotherapy and EGFR-TKI alone groups; the median PFS in the two groups was 12.6 and 12.4 months, respectively (**Figure 3A**), with no significant differences (log-rank *P*=0.9434; HR, 1.014; 95% CI, 0.6871–1.497; **Figure 3A**).

**Figure 4:**
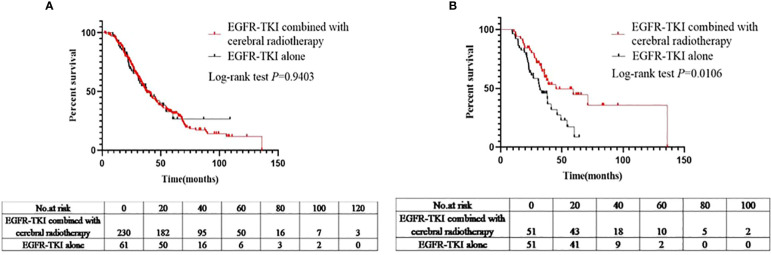
**(A)** Comparison of OS between the EGFR-TKIs combined with cerebral radiotherapy group and EGFR-TKI alone group before PSM. **(B)** Comparison of OSbetween the EGFR-TKIs combined with cerebral radiotherapy group and EGFR-TKI alone group after PSM.

The median OS for patients in the EGFR-TKI+craniocerebral radiotherapy and EGFR-TKI alone groups was 37.6 and 38.1 months, respectively (**Figure 4A**), with no significant differences observed (log-rank *P*=0.9403; HR, 1.015; 95% CI, 0.6935–1.484; **Figure 4A**).

### Univariable and multivariable analyses of covariable with the iPFS, PFS, and OS results

The univariate analysis of covariables with the iPFS (**Table 2**) and PFS (**Table 3**) results showed that the effect of sex, age, smoking status, and *EGFR* mutation was not statistically significant. Multivariate analysis showed that only lung-molGPA was an independent predictive factor (*P*=0.002 for both).

**Table 2:** Univariable and multivariable analyses of covariables associated with ipfs.

Variable	Univariable Analysis			Multivariable Analysis		
	HR	95% CI	P	HR	95% CI	P
Age(y)						
>60 vs ≤60	0.9001	0.6025 to 1.345	0.6071			
Sex						
Female vs male	1.127	0.7664 to 1.656	0.5441			
Smoking Status						
Never vs current/former	0.8911	0.5869 to 1.353	0.5885			
EGFR Mutation						
Exon 19	0.8885	0.6055 to 1.304	0.5459			
Exon 21	0.8667	0.5930 to 1.267	0.4600			
First-line TKI therapy						
Yes vs no	0.9431	0.6448 to 1.379	0.7625			0.938
Lung-molGPA						
1-2 vs 2.5-4	0.5266	0.3513 to 0.7895	0.0019	0.552	0.377 to 0.809	0.002
Group						
EGFR-TKIs combined with cerebral radiotherapy group vs EGFR-TKI alone group	1.376	0.7661 to 2.472	0.2855			0.288

EGFR, epidermal growth factor receptor; TKI, tyrosine kinase inhibitors.

**Table 3:** Univariable and multivariable analyses of covariables associated with pfs.

Variable	Univariable Analysis			Multivariable Analysis		
	HR	95% CI	P	HR	95% CI	P
Age(y)						
>60 vs ≤60	1.184	0.8660 to 1.618	0.2901			0.218
Sex						
Female vs male	0.9825	0.7240 to 1.333	0.9096			
Smoking Status						
Never vs current/former	1.000	0.7057 to 1.418	0.9988			
EGFR Mutation						
Exon 19	0.8892	0.6572 to 1.203	0.4466			0.535
Exon 21	1.044	0.7763 to 1.405	0.7746			0.936
First-line TKI therapy						
Yes vs no	0.9982	0.7409 to 1.345	0.9905			
Lung-molGPA						
2.5-4 vs 1-2	0.6163	0.4538 to 0.8370	0.0019	0.628	0.466to 0.846	0.002
Group						
EGFR-TKIs combined with cerebral radiotherapy group vs EGFR-TKI alone group	0.9860	0.6680 to 1.455	0.9434			

The univariate and multivariate analyses of the covariables are shown in **Table 4**. Results of the univariate analysis showed that the effects of sex, age, and smoking status were not statistically significant. Multivariate analysis showed that *EGFR* exon 19 deletion, lung-molGPA, and the start date of EGFR-TKIs were independent predictive factors (*P*=0.082, HR, 1.479, 95% CI, 0.952–2.297; *P ≤* 0.001, HR, 1.873, 95% CI, 1.408–2.491; *P*=0.9403; *P*=0.037, HR, 0.736, 95% CI, 0.552–0.981; respectively).

**Table 4:** Univariable and multivariable analyses of covariables associated with os.

Variable	Univariable Analysis			Multivariable Analysis		
	HR	95% CI	P	HR	95% CI	P
Age(y)						
>60 vs ≤60	1.102	0.8194 to 1.483	0.5200			
Sex						
Female vs male	1.103	0.8230 to 1.479	0.5112			
Smoking Status						
Never vs current/former	1.258	0.8864 to 1.784	0.1991			
EGFR Mutation						
Exon 19	1.372	1.031 to 1.825	0.0302	1.479	0.952 to 2.297	0.082
Exon 21	1.223	0.9199 to 1.627	0.1657	1.146	0.760 to 1.790	0.480
First-line TKI therapy						
no vs Yes	0.6982	0.5231 to 0.9320	0.0148	0.736	0.552 to 0.981	0.037
Lung-molGPA						
1-2 vs2.5-4	2.024	1.502 to 2.727	<0.0001	1.873	1.408 to 2.491	<0.001
Group						
EGFR-TKIs combined with cerebral radiotherapy group vs EGFR-TKI alone group	1.015	0.6935 to 1.484	0.9403			

### Survival analysis after PSM

According to the results of the Chi-squared test (**Table 1**), age, time of targeted therapy application, and lung-molGPA score had different constituent ratios (*P*=0.0106; *P*=0.0017; *P*=0.0262, respectively) between the groups. Combined with univariable and multivariable analyses (**Tables 2**–**4**), sex, age, TNM stage, lung-molGPA score, and the time of targeted application affected the prognosis of patients. We performed 1:1 PSM to eliminate the influence of these factors on the results. The group indicator was whether it was combined with craniocerebral radiotherapy, and the predictors were sex, age, TNM stage, lung-molGPA score, and the time of targeted application. The predictors were defined as follows: gender classification, i.e., males and females; age, defined as the years at the time of initial diagnosis; TNM staging, classified as III or IV; and the time of targeted application, the time when the patients was first administered targeted drugs. The tendency score was calculated using logistic regression analysis. The nearest neighbor algorithm (caliper: 0.2) was used to match the propensity score.

This study enrolled 102 patients, with 51 patients in each treatment group. The median age at diagnosis was 54 years (range, 28–81 years). Prior to the PSM analysis, women (57, 55.9%) and non-smokers (77, 75.5%) accounted for majority of the patients. Mutations in *EGFR* exons 19 and 21 were detected in 45.1% (46/102) and 48.0% (49/102) of patients, respectively. The majority of lung-molGPA scores of the patients were in the 2.5–4 group (77,75.5%).

After the PSM analysis, 57 deaths were recorded. The median follow-up duration was 40.3 months (interquartile range, 34.5–63.8) months. The median OS, iPFS, and PFS was 38.1 months, 13.9 months, and 13.8 months, respectively (**Figures 1A–C**). There was no difference in the OS between groups with mutations in exons 19 and 21 (35.7 months vs. 38.1 months; log-rank *P*=0.7289; HR, 0.9090; 95% CI, 0.5299–1.559; **Figure 1D**).

Sixty patients had intracranial progression after treatment with EGFR-TKIs (EGFR-TKIs+craniocerebral radiotherapy group, 36; and EGFR-TKI alone group, 24). Similarly, the median iPFS in the EGFR-TKI+craniocerebral radiotherapy group and EGFR-TKI alone group was 14.7 and 8.9 months, respectively (**Figure 2B**), with significant differences observed (log-rank *P*=0.0220; HR, 0.4278; 95% CI, 0.2069–0.8848; **Figure 2B**).

Ninety-nine patients developed disease progression after using EGFR-TKIs (EGFR-TKIs+craniocerebral radiotherapy group, 45; and EGFR-TKI alone group, 44). The median PFS in the EGFR-TKIs+craniocerebral radiotherapy group and EGFR-TKI alone group was 16.2 and 11.9 months, respectively (**Figure 3B**); with significant differences observed between the groups (*P*=0.0324; HR, 0.5524; 95% CI, 0.3208–0.9515; **Figure 3B**).

Furthermore, after PSM analysis, the median OS of the EGFR-TKI+craniocerebral radiotherapy and EGFR-TKI alone groups were 45.3 and 32.1 months, respectively, with a significant difference observed between the regimens (log-rank, *P*=0.0106; HR, 0.4924; 95% CI, 0.2861–0.8476; **Figure 4B**).

## Discussion

In our study, iPFS, PFS, and OS in the EGFR-TKI combined with craniocerebral radiotherapy group were not significantly improved as compared to those who were administered TKIs alone prior to PSM. A variety of clinical and pathological factors can affect the prognosis of *EGFR*-mutant lung adenocarcinoma with brain metastases. Therefore, we speculate that the heterogeneity of these factors between the two groups contributed to the lack of statistical difference in survival analysis (8, 12, 25–27). We initially used a Chi-squared analysis to analyze whether there were differences in the general clinical characteristics between the two groups. The results indicated that differences in age, timing of EGFR-TKI administration, and lung-molGPA existed between the two groups. Univariable and multivariable analyses of covariables associated with iPFS, PFS, and OS also showed that lung-molGPA was independently associated with improved iPFS, PFS, and OS. Therefore, insignificant difference in the survival analysis between the two groups before PSM may be due to uneven clinical features, especially those affected by lung-molGPA. Lung-molGPA reportedly provides a better predictive value, suggesting that lung-molGPA can comprehensively and accurately reflect the prognosis of NSCLC complicated by brain metastases (24, 28–30). These also suggest the importance of individualized therapy for craniocerebral radiotherapy (12, 31–33). Moreover, most previous retrospective studies have shown that EGFR-TKI concurrently administered with radiotherapy was more effective than treatment with EGFR-TKI alone, which was not consistent with the results of our study (10, 19, 34, 35). However, most of these studies did not consider these variables during clinical decision making.

To further elucidate the role and value of craniocerebral radiotherapy in the treatment of patients with lung cancer, we used the PSM method to minimize the impact of these factors, especially the impact of differences in lung-molGPA, which could more accurately compare the effects of EGFR-TKIs combined with craniocerebral radiotherapy and EGFR-TKI alone (36–38). There was no statistical difference in the clinical features between the two groups after matching. After PSM, EGFR-TKIs combined with craniocerebral radiotherapy improved iPFS, OS, and PFS as compared to EGFR-TKIs alone. To the best of our knowledge, our study is the first to use PSM to compare the effects of EGFR-TKIs combined with craniocerebral radiotherapy and EGFR-TKIs alone on the prognosis of patients with *EGFR*-mutant lung adenocarcinoma with brain metastases. Our results suggest that EGFR-TKIs combined with craniocerebral radiotherapy not only increases the iPFS of the patients but also translates the prolongation of PFS into an improvement in OS. A retrospective study involving a small sample size showed that the combination of craniocerebral radiotherapy and EGFR-TKIs could improve iPFS, while OS and PFS were not significantly prolonged, when compared with EGFR-TKIs alone (19, 22, 23). In another study, the median iPFS and OS of the EGFR-TKIs group combined with the craniocerebral radiotherapy group were significantly longer than those in the EGFR-TKI alone group (39).

Our results emphasize the importance of performing craniocerebral radiotherapy as a treatment for these patients. However, the appropriate timing of craniocerebral radiotherapy requires further investigation. A study showed that in NSCLC patients with *EGFR* mutations, pre-radiotherapy showed better results, with a median OS of 30 months for patients who received pre-WBRT and 25 months for those who received pre-EGFR-TKIs (23). In patients with *EGFR*-mutant NSCLC and brain metastases, craniocerebral radiotherapy followed by EGFR-TKIs improved iPFS compared with upfront EGFR-TKI, showing that the delay or lack of craniocerebral radiotherapy was associated with a poor PFS (8). Our study also enrolled patients who received craniocerebral radiotherapy after EGFR-TKI resistance; however, this did not change the results which stated that the iPFS, PFS, and OS of the combined treatment regimen group were longer than those of the EGFR-TKI alone group. Combining the results of previous studies with our results (including the BRAIN clinical trial), we can suggest that patients with better clinical conditions and good prognosis can be treated with EGFR-TKI alone first, which can reduce the long-term adverse events caused by craniocerebral radiotherapy and improve the quality of life of patients (16, 40). For these patients, the progression of brain metastases after EGFR-TKI treatment can be treated with craniocerebral radiotherapy to obtain longer craniocerebral local control and improve the survival prognosis of the patients. However, for patients with worse clinical conditions, especially those with lower lung-molGPA scores, craniocerebral radiotherapy can be combined with EGFR-TKIs concurrently to increase local control. Our results also suggest that prolonged iPFS translates into a better OS, due to which patients can have a better prognosis. Therefore, the combination of craniocerebral radiotherapy and EGFR-TKIs should not be a one-size-fits-all model, and should instead be considered comprehensively to provide patients with the best individualized treatment plan. The formulation of craniocerebral radiotherapy strategies should be based on a comprehensive evaluation of the patient’s lung-molGPA score while making treatment decisions (12, 41, 42).

This study had some limitations. First, it was a single-center, retrospective study with a limited sample size, and thus could have had a selection bias. However, although we used the PSM method to reduce the interference of some potential factors, the individual factors may have still affected our results. Therefore, a multi-institutional, prospective study with a large sample size is required to confirm the findings of this study. Subsequently, the relevant experiments were designed to provide theoretical support that EGFR-TKIs combined with craniocerebral radiotherapy is a superior treatment option, as compared to EGFR-TKIs alone, for future work at the experimental level.

## Conclusion

Our study showed that in patients with *EGFR*-mutant lung adenocarcinoma and brain metastases, craniocerebral radiotherapy plays an important role in improving the survival and prognosis, indicating that EGFR-TKIs combined with craniocerebral radiotherapy might be a better therapeutic option for this patient population. Furthermore, a combination strategy should be developed for individualized treatment plans based on various prognostic parameters.

## Data availability statement

The raw data supporting the conclusions of this article will be made available by the authors, without undue reservation.

## Ethics statement

The studies involving human participants were reviewed and approved by the Ethics Committee of Shandong Cancer Hospital and Institute. Written informed consent for participation was not required for this study in accordance with the national legislation and the institutional requirements.

## Author contributions

Conceptualization, GD, and XT. Methodology, GD. Software, GD. Validation, GD and XT. Formal analysis, GD and ZL. Resources, GD. Data curation, GD, XT, YL and YZ. Writing—original draft preparation, GD. Writing—review and editing, GD and ZL. Supervision, JL and ZL. All authors contributed to the article and approved the submitted version.
