# Supplementary material for: Effect of EGFR-TKIs combined with craniocerebral radiotherapy on the prognosis of EGFR-mutant lung adenocarcinoma patients with brain metastasis: A propensity-score matched analysis
Source: Front Oncol. 2023 Feb 9;13:1049855. doi: 10.3389/fonc.2023.1049855 (PMC9948088; doi:10.3389/fonc.2023.1049855)
Supplement: Supplementary file 1 [file DataSheet_1.docx]

**Highlights:**

- The incidence of brain metastasis in lung adenocarcinoma patients with EGFR mutations is slightly higher than that in patients with wild-type EGFR, and EGFR-TKIs alone were effective but their therapeutic effect was limited
- IPFS, PFS, and OS in the EGFR-TKI combined with craniocerebral radiotherapy group were not significantly improved as compared to those who were administered TKIs alone prior to PSM.
- We use PSM method to eliminate the impact of potential confounding factors on the results, which compared with EGFR-TKI alone, EGFR-TKIs combined with craniocerebral radiotherapy may improve iPFS, PFS and OS.
- The participation of craniocerebral radiotherapy strategies should be based on a comprehensive evaluation of the patient's lung-molGPA score while making individualized treatment plan.
